# Self-Compassion and Parenting in Mothers and Fathers with Depression

**DOI:** 10.1007/s12671-016-0528-6

**Published:** 2016-05-03

**Authors:** L. Psychogiou, K. Legge, E. Parry, J. Mann, S. Nath, T. Ford, W. Kuyken

**Affiliations:** School of Psychology, University of Exeter, EX4 4QG Exeter, UK; The Oxford Institute of Clinical Psychology Training, University of Oxford, Oxford, UK; Medical School, University of Exeter, Exeter, UK; Department of Psychiatry, University of Oxford, Oxford, UK

**Keywords:** Depression, Internalizing, Fathers, Self-compassion, Parenting

## Abstract

Depression in parents impairs parenting and increases the risk of psychopathology among their children. Prevention and intervention could be informed by knowledge of the mechanisms that break the inter-generational transmission of psychopathology and build resilience in both parents and their children. We used data from two independent studies to examine whether higher levels of self-compassion were associated with better parenting and fewer emotional and behavioral problems in children of parents with a history of depression. Study 1 was a pilot trial of mindfulness-based cognitive therapy that included 38 parents with recurrent depression. Study 2 was a longitudinal study that consisted of 160 families, including 50 mothers and 40 fathers who had a history of depression. Families were followed up approximately 16 months after the first assessment (time 2; *n* = 106 families). In both studies, self-compassion was assessed with the Self-Compassion Scale. Parents reporting higher levels of self-compassion were more likely to attribute the cause of their children’s behavior to external factors, were less critical, and used fewer distressed reactions to cope with their children’s emotions. Parents’ self-compassion was longitudinally associated with children’s internalizing and externalizing problems, but these associations became nonsignificant after controlling for child gender, parent education, and depressive symptoms. Future larger scale and experimental designs need to examine whether interventions intended to increase self-compassion might reduce the use of negative parenting strategies and thereby the inter-generational transmission of psychopathology.

## Introduction

The lifetime prevalence of depressive disorder is between 10 and 15 % (Lépine and Briley 2011) and is associated with significant impairments in many domains of functioning. Depression in mothers and fathers is associated with emotional and behavioral problems in their children (e.g., Connell and Goodman 2002; Goodman 2007; Goodman et al. 2011; Ramchandani et al. 2005; Weitzman et al. 2011). Depression may undermine parents’ capacity to parent and the quality of parent–child interactions (Goodman 2007; Goodman and Gotlib 1999; Ramchandani and Psychogiou 2009). Mothers with depression may express fewer positive emotions, have less child-oriented and more parent-oriented concerns (Dix et al. 2004), and exhibit behavior toward their children that is more hostile and less positive and engaging compared to mothers without depression (e.g., Lovejoy et al. 2000). Depressed fathers similarly may display more negative and less positive parenting behaviors (Nath et al. 2015; Wilson and Durbin 2010). For example, depressed fathers reported that they smacked their 12-month-old children more (41 vs 13 %) (Davis et al. 2011) and spent less time with their children on positive activities (Paulson et al. 2006), compared to fathers without depression. Although a link between parental depression and parenting is established, the mechanisms that contribute to impaired parenting remain poorly understood and understudied. Therefore, a key gap in the literature is an understanding of the mechanisms that potentially underpin depression and parenting difficulties. An emergent literature (e.g., Diedrich et al. 2014; Krieger et al. 2013; Kuyken et al. 2010) on the importance of self-compassion in depression suggests a promising avenue of research.

According to cognitive theory, people at risk for depression tend to have attentional biases to negative material, negative beliefs, upsetting images and memories, ruminative thinking, and avoidant behaviors that can be easily triggered and escalate into depression (Beck and Haigh 2014). Finding ways to manage these cognitive tendencies may enable those at risk for depression to be more resilient, including their role as parents. Self-compassion is typically defined as recognizing and being open to one’s suffering, being kind toward oneself, and seeing one’s experience as part of the larger human experience (Barnard and Curry 2011; Feldman and Kuyken 2011; Neff 2003a). It provides an alternative to the negative thinking, avoidance, and rumination that characterize depression. Self-compassion potentially enables a person to respond to negative thoughts and feelings with kindness, equanimity, and patience. This helps them de-center from negative thinking and feelings, which in turn prevents the escalation and maintenance of depressive thinking and behavior (Feldman and Kuyken 2011). There is a growing literature that suggests that self-compassion may be an adaptive strategy for managing the types of negative thoughts and feelings described above (Barnard and Curry 2011). There is now also evidence that among people with a history of depression, self-compassion may help them to overcome problematic patterns of avoidance and rumination (Diedrich et al. 2014; Krieger et al. 2013).

Self-compassion has also been found to be associated with intrinsic motivation, health behaviors like exercise, body image, personal responsibility, more caring and supportive relationship behavior (Neff et al. 2007), as well as their bio-behavioral correlates (Breines et al. 2014; Klimecki et al. 2013a, b). Self-compassion was also impaired among people suffering depression and was associated with differences in emotion regulation (Diedrich et al. 2014; Krieger et al. 2013). Interventions like mindfulness-based cognitive therapy that cultivate self-compassion can prevent depressive relapse/recurrence, and there is some evidence that outcomes are mediated through learning self-compassion (Kuyken et al. 2010). Thus, it is likely that self-compassion may also be linked to parenting behaviors. For example, self-compassion may enable parents to recognize, allow, and de-center from negative thoughts so they can be more aware of and less reactive to negative thoughts. This may increase their ability to respond sensitively to their children’s needs (Kabat-Zinn and Kabat-Zinn 1998). Parents with greater self-compassion might increase their capacity to respond adaptively to triggers and decrease the frequency of over-learned automatic patterns of thinking and behaving. These processes may manifest as an increased ability to respond to the challenges of parenting in ways that are more sensitive and resilient. For example, a woman trying unsuccessfully to console her crying infant during the night might have the self-judging thought “I am a bad mother.” In depression, this might spiral into a sense of helplessness and hopelessness. However, an individual with greater self-compassion might respond to the same thought with “wait, where did that come from, that’s a very negative thought, I must be very tired and need some sleep. I’ll ask my partner if he can do the next feed and take some naps with my child tomorrow.” Parents who embody self-compassion may also model resilience for their children. It is possible that children learn self-compassion expressed by their parents, a hypothesis that would be interesting to test. Thus, for example, a child who sees his or her parent respond with self-compassion may learn to respond in the same way through observational learning. Another possibility is that self-compassion may be translated into more positive parenting, which in turn may protect children from the negative effects of parental depression. Parents who score high on self-compassion may display more responsiveness and warmth. Therefore, it is likely that parents’ self-compassion is linked both to parenting and children’s outcomes.

The preschool years are a key developmental period for several reasons. The effects of depression might be more evident on young children given young children’s dependence on their parents. Preschool children have to meet key goals (e.g., learn to regulate their emotions, behave appropriately in structured settings, establish and maintain positive peer relationships) to make a successful transition to school. Depression may limit parents’ capacity to model and scaffold the necessary skills for their children’s attainment of these goals. Furthermore, elevated levels of emotional and behavioral problems in the child may tax the insufficient inner resources of the depressed parent, and the parent–child dyad may then become entrenched in a negative cycle of interaction that reinforces each other’s negative behavior (Patterson 1982). Childhood emotional and behavioral problems can be persistent and predict adverse future outcomes including poor academic achievement, aggressive and antisocial behavior, as well as adult psychiatric disorders (Campbell 1995; Egger and Angold 2006; Kim-Cohen et al. 2003).

In this paper, we examined data from two studies to explore whether self-compassion may be associated with internal states related to parenting (more adaptive attributions), emotional regulation in the face of challenges (coping well with children’s negative emotions), expressed emotion (more positive and fewer critical comments about their children), and parents’ ability to respond to their children sensitively. In addition, we explored whether there was an association between self-compassion among parents and lower levels of emotional and behavioral problems that they reported in their children.

## Study 1

### Method

#### Participants

The sample consisted of 36 mothers and 2 fathers who reported three or more major depressive episodes and were currently either in full or partial remission. The parents’ mean age was 36.2 years (*SD* = 5.1). In total, 71 % (*n* = 27) of participants were married, 8 % (*n* = 3) were cohabitating, 11 % (*n* = 4) were divorced, 5 % (*n* = 2) were separated, and 5 % (*n* = 2) were single. The majority of participants (58 %, *n* = 22) had a university degree or professional qualification, 32 % (*n* = 12) had a college or vocational qualification, and 8 % (*n* = 3) had some school qualification. Their children’s age ranged from 2 to 6 years (*M* = 4.1, *SD* = 1.3); 15 (39.5 %) were girls. Only one child per family participated in the study. In addition to a history of three or more depressive episodes, inclusion criteria were current remission from depression, age 18 or over, and at least one child aged between 2 and 6 years old. Exclusion criteria were current or a history of psychosis, current substance dependence, organic brain damage, persistent longstanding interpersonal difficulties, safe-guarding concerns about children in the family, antisocial behavior or persistent self-harm, and receiving psychological therapy.

#### Procedure

Recruitment took place through GP practices and advertisement at health and school services. Individuals participated in a screening interview and eligibility was confirmed using the Structured Clinical Interview for DSM-IV (SCID; Gorman et al. 2004) to assess for depression (including identification of remission status and number of episodes) and comorbidity. Parental consent was obtained for all parents and children to take part in the study, and at this point, all parents completed the measures described below. Randomization then took place with parents randomized to a mindfulness-based cognitive therapy course for parents or to usual care. Parents completed all measures at baseline, prior to any intervention. Further detail about the procedure for the pilot trial is provided elsewhere (Mann et al., under review).

#### Measures

##### Parents’ Self-Compassion

The Self-Compassion Scale (SCS; Neff 2003b) consists of 26 items, each rated on a five-point Likert scale (1 = almost never to 5 = almost always), which has excellent reliability and validity (Neff et al. 2007). The measure correlates significantly with measures of self-esteem (*r* = 0.59), self-acceptance (*r* = 0.62), and self-determination (*r* = 0.43), and its test-retest reliability over a 3-week interval is *r* = 0.93 (Neff 2003b). Higher scores indicate greater self-compassion. We used the SCS because it addresses our definition of self-compassion, and while it has some psychometric limitations, it has shown adequate reliability and validity and is the best available measure (Williams et al. 2014).

##### Parents’ Depression

Parents’ depression was assessed with the Structured Clinical Interview for DSM-IV (SCID; Gorman et al. 2004). A trained researcher asked parents questions to assess for current depression and any past history of depression. The SCID for DSM-III-R has been compared to gold standard diagnoses (in which all available medical information was used, in addition to information from the SCID), and it demonstrated 91 % specificity and 84 % sensitivity (Basco et al. 2000). Its inter-rater reliability for diagnosing major depressive disorder is *k* = 0.66 (Lobbestael et al. 2010).

##### Parents’ Depressive Symptoms

The Beck Depression Inventory Second Edition (BDI-II; Beck et al. 1996) is a 21-item questionnaire structured around cognitive and affective symptoms. Scores 0–13 indicate minimal depression, 14–18 mild depression, 19–29 moderate depression, and 30–63 severe depression. The scale has excellent reliability (a = 0.94) and convergent validity. Significant correlations that range from −0.19 to −0.65 have been found between the BDI score and measures of general perceptions of health and functioning in the expected direction (Arnau et al. 2001).

##### Parents’ Sensitive Responding

Parent–child pairs took part in three tasks: (1) free play with no specific instructions, (2) a highly structured Lego task designed to be hard for the child to complete unaided, and (3) a tidy up task where the parent and child tidied all toys away. Each task lasted for 5 min. Interactions were video-recorded and were coded later using the Coding of Attachment-Related Parenting (CARP; Matias et al. 2006). The CARP is based on attachment theory and assesses the quality of parent–child interaction styles. Parents’ sensitive responding was rated on a seven-point scale ranging from 1 = unresponsive/insensitive parent to 7 = responsive/sensitive parent. An overall score of sensitivity was created by adding the scores from all three tasks. The CARP’s sensitivity scale has been demonstrated to significantly correlate with the security of children’s attachment as measured by a story stem procedure, *rs* = 0.20, *ps* < 0.5 (O’Connor et al. 2013). The stability of ratings over a year has been demonstrated to be satisfactory, *r* = 0.66, while inter-rater reliability has been demonstrated to be good with intraclass correlations of 0.73 (O’Connor et al. 2013).

Researchers were trained by the developers of the measure (National Academy of Parenting Research (NAPR), Kings College London). Prior to coding the study data, researchers coded gold standard videos to check the reliability of their coding. Inter-rater reliability as assessed using the intraclass correlation coefficient (ICC) calculated using a two-way random absolute agreement, which was excellent (ICC > 0.8 for both coders). Raters attended weekly meetings to avoid coding drift. Coders were blind to participants’ scores on the measures of interest at the time of coding.

##### Parents’ Attributions of Their Children’s Behavior

Parental attributions concerning their children’s behavior were assessed using a measure of parental attributions, developed by Dadds, Scott, and Woolgar at the National Academy of Parenting Research (NAPR, UK) from earlier work assessing the impact of parental attributions on parenting and children’s behavior (Dadds et al. 2003). It uses a semistructured interview consisting of six ambiguous scenarios of children’s behaviors and asks parents to imagine their child in each scenario. The current study focused on the parents’ answers to two questions: “How would you describe the behavior” and “why is he/she acting like that?” Answers were allocated two codes according to the valence (defined below) and attribution that parents reported in relation to their child’s behavior. The code assigned for valence refers to the parents’ interpretation of the child’s behavior and was coded on a five-point Likert scale, ranging from (+2/positive) to (−2/negative). A score of zero indicates a neutral judgment.

The separate code assigned to attribution refers to the cause the parent ascribed to their child’s behavior. An internal attribution ascribes the behavior to personal dispositions, traits, or abilities (commonly global and stable over time) whereas an external attribution ascribes the causes to situational demands and environmental constraints (commonly situation specific and transient). Attribution was coded on a five point-Likert scale; the highest score (+2) represented an internal and the lowest code (−2) an external attribution. A zero score indicates a mixed attribution.

The scores from these two scales were then combined to calculate total scores for positive and negative attributions, respectively. The “negative attributions” score aimed to quantify the tendency of parents to attribute children’s negative behavior to internal and often stable characteristics of the child. It was calculated by adding the attribution scores from each scenario in which the valence was rated negatively. The total positive attribution score was similarly calculated by adding the attribution scores from each scenario in which the valence was rated positively. This aimed to estimate the extent to which childhood behaviors were attributed to external demands and environmental factors that the child was reacting to versus internal, longstanding character traits.

The attribution measure coder was trained and assessed for inter-rater reliability (prior to coding the study data) by NAPR. The ICC was calculated using a two-way random absolute agreement. The coder achieved ICC = 0.9 for valence and ICC = 0.7 for the attribution domain. Coders were blind to participants’ scores at the time of coding.

### Data Analyses

We first examined correlations of self-compassion with sensitive responding and attributions. Hierarchical linear regressions were undertaken to test the association between parents’ self-compassion (independent variable) and parenting behaviors (attributions and sensitive responding). We controlled for the effects of confounding variables including children’s gender and parents’ education and current depressive symptoms. Child gender was included as the literature indicates some differences in parenting between boys and girls. For example, Maniadaki et al. (2005) found that parents of preschool children attributed more intentionality for hyperactive/ impulsive behaviors to boys than girls. Parenting difficulties are also more likely to occur if parents suffer from depression (e.g., Lovejoy et al. 2000; Wilson and Durbin 2010) and are less educated (Davis-Kean 2005).

Children’s gender, parents’ education, and current depressive symptoms were entered at step 1 and self-compassion at step 2. Although the sample was small, hierarchical regressions were used because we were less interested in how much variance in the outcome was explained by all the predictors. In addition, the residuals were normally distributed and there were no outliers. Finally, we calculated effect sizes using adjusted *R*^2^ which is a better indicator of the effects on the population.

## Results

Range, means, and standard deviations for all measures for study 1 are presented in Table 1. The overall self-compassion scale correlated with both positive (*r* = −0.45, *p* < 0.01) and negative attributions (*r* = −0.42, *p* < 0.05). There were no significant associations between self-compassion and sensitive responding (*r* = 0.29, *p* = 0.127). Table 2 shows that when controlling for child gender, parent education, and depressive symptoms, the associations between self-compassion and positive (*β* = −0.45, *p* < 0.05) and negative (*β* = −0.42, *p* < 0.05) attributions remained significant. The effect size for positive attributions was *f*^2^ = 0.25, and for negative attributions, it was *f*^2^ = 0.19, which indicate medium to large effects.

**Table 1:** Range, means, and standard deviations for study variables (study 1)

	Number	Range	*M* (*SD*)
Self-compassion	33	37–88	61.30 (13.26)
Depressive symptoms	38	0–32	11.21 (9.17)
Parental sensitive responding across three tasks	34	6–19	13.18 (3.42)
Positive attributions	37	(−4)–(5)	0.38 (2.45)
Negative attributions	37	(−5)–(2)	−1.05 (1.55)

**Table 2:** Hierarchical linear regressions showing the associations of parents’ self-compassion with parenting after controlling for children’s gender and parents’ education and depressive symptoms (study 1)

	Positive attributions			Negative attributions			Sensitive responding		
	*N*	*ΔR* ^2^	*β*	*N*	*ΔR* ^2^	*β*	*N*	*ΔR* ^2^	*β*
Step 1	31	0.16		31	0.09		30	0.15	
Children’s gender (1 = female, 2 = male)			−0.09			−0.29			0.37
Parents’ education (0 = no qualification or diploma, 1 = degree or postgraduate)			0.31			−0.05			0.24
Parents’ depressive symptoms			0.27			0.19			−0.13
Step 2		0.17^a^			0.15^a^			0.07	
Children’s gender (1 = female, 2 = male)			−0.07			−0.27			0.35
Parents’ education (0 = no qualification or diploma, 1 = degree or postgraduate)			0.31			−0.05			0.26
Parents’ depressive symptoms			0.09			0.02			−0.03
Parents’ self-compassion			−0.45^a^			−0.42^a^			0.29

^a^<0.05

## Discussion

Contrary to the study hypothesis, self-compassion was not significantly associated with sensitive responding. However, this may be due to the relatively small sample size. Study 1 suggests that parents who had higher levels of self-compassion were more likely to attribute the cause of their children’s behavior to external factors (such as situational influences, demands, or constraints) for both positive and negative situations. It is possible that self-compassion, which includes seeing experiences, both positive and negative as part of the wider human condition leads parents with greater self-compassion to explain their children’s behavior as responses to external factors rather than to innate character traits of their child. This is an intriguing hypothesis for further research. Study 2 sought to examine whether consistent pattern of associations appear in complementary parenting behaviors in a larger sample of mothers and fathers.

## Study 2

### Method

#### Participants

Data were drawn from the fathers in focus study, a longitudinal study that investigates the links of parental depression with parenting and child outcomes. In total, 160 families participated in the study at time 1 while 66 % of families (*n* = 106) were visited again 16 months (*M* = 16.2, *SD* = 3.7) after the first assessment. There were no significant differences in levels of depressive symptoms at baseline between fathers, *t*(155) = −1.46, *p* = 0.15, and mothers, *t*(142) = 0.10, *p* = 0.92, who stayed and parents who refused to participate at time 2. Inclusion criteria were biological parent, regular contact with the child, ability to read and speak English, consent to allow home visit, and children aged 3–5 years. Exclusion criteria were medical and neurological disorders, language, and cognitive delays in the child.

#### Procedure

Fathers were recruited via nurseries, community centers, and posters displayed at sports and community centers. In addition, we identified eligible fathers with current depression (major depressive episode in the last month) or a history of depression (major depressive episode at any other time) diagnosed by a clinician by electronic case record searchers in ambulatory care. Fathers received an information leaflet and the Patient Health Questionnaire (PHQ-9; Spitzer et al. 1999), which is a screening questionnaire for depression. In total, we sent out 1277 invitation letters/PHQ-9 and received back 544. Of those who returned the questionnaire 319 agreed to be contacted about the follow-up study. Of the 319, 160 took part, 68 refused once contacted, 52 did not respond to messages, and 39 were excluded because they did not meet the study’s inclusion criteria.

While the partners’ participation was not among the inclusion criteria, partners were also invited to take part. Fathers who gave their written consent to participate were contacted by a trained researcher at Masters Level and were asked if they could be seen at home. At this stage, their partners were also asked if they were willing to facilitate the study. At the beginning of the assessment, a trained psychologist provided information about the study and asked each parent to sign a consent form. Each parent independently completed the measures described below. Each parent was also independently asked questions about current (in the last month) and past history of depression using the Structured Clinical Interview for DSM-IV (SCID; Gorman et al. 2004). The term “research diagnosis of depression” was used to indicate that depression was assessed in a research rather than a clinical setting. In the present study, 40 fathers and 50 mothers met the diagnostic criteria for a major depressive Episode according to the SCID (SCID; Gorman et al. 2004) at time 1.

#### Measures

##### Parents’ Self-Compassion

As in study 1, this was measured with the SCS (Neff 2003b), which was completed by both mothers and fathers at time 1.

##### Parents’ Depressive Symptoms

The Patient Health Questionnaire (PHQ-9; Spitzer et al. 1999) is a screening questionnaire for depression based on DSM-IV diagnostic criteria. It consists of nine items, each rated on a four-point scale ranging from 0 = not at all to 3 = nearly every day. Scores of 5, 10, 15, and 20 indicate mild, moderate, moderately severe, and severe depression. Studies have shown agreement between PHQ-9 diagnosis and diagnosis made by independent mental health professionals (overall accuracy 85 %, sensitivity 75 %, and specificity 90 %). The measure also correlates significantly with measures of health care use and impairment (Spitzer et al. 1999). The PHQ-9 was completed by both mothers and fathers at times 1 and 2.

##### Parents’ EE Toward Their Children

Parental expressed emotion (EE) was measured at times 1 and 2 using the Preschool Five Minute Speech Sample (PFMSS; Daley et al. 2003). Each parent was asked to talk for 5 min about their thoughts and feelings toward their child and their relationship over the last 6 months. The P-FMSS has the following categories: initial statement, relationship, warmth, and frequency count of positive and critical comments. We focused on the continuous measures of positive and critical comments to examine whether these EE contrasting constituents correlated differently with the self-compassion subscales. The coding scheme has satisfactory reliability and validity (Daley et al. 2003). In this study the inter-rater reliability was examined in 40 speech samples (20 mothers and 20 fathers) and was high for both critical (ICC = 0.9) and positive comments (ICC = 0.9). The coders were blind to parents’ characteristics.

##### Parents’ Coping with Their Children’s Negative Emotions

The Coping with Children’s Negative Emotions Scale (CCNES; Fabes et al. 1990) consists of 12 scenarios. Parents rate on a seven-point scale (from 1 = very unlikely to 7 = very likely) how likely they are to respond to each scenario in six different ways. The study focused on the extent to which the parent experiences distress by their child’s display of negative emotions (“distressed reactions”) and helps the child to solve the problem (“problem-focused reactions”). Both the distressed reactions and problem-focused reactions scale have good internal consistency (*a* = 0.79 and *a* = 0.80, respectively) (Eisenberg and Fabes 1994). The CCNES was completed by both parents at times 1 and 2.

##### Children’s Internalizing (Emotional) and Externalizing (Behavioral) Problems

The Child Behavior Checklist for ages 1½ to 5 (Achenbach and Rescorla 2000) was used to measure children’s internalizing and externalizing problems at time 1 and time 2 if they remained within that age group. Otherwise, parents completed the CBCL for ages 6 to 18 for those children who were 6 years old and above at time 2. Responses were rated on a three-point scale ranging from 0 = not true to 2 = very true or often true. At time 2, standardized scores for each scale were created to allow analysis from both versions of the CBCL as follows. For each version of the scale, internalizing and externalizing scale scores at time 2 were transformed into separate z-scores. We then merged corresponding z-scores into each of two new variables representing scale-invariant, standardized values of internalizing and externalizing problems respectively at time 2.

### Data Analyses

Pearson’s correlations were computed to examine concurrent and longitudinal associations of self-compassion with study variables. Hierarchical linear regressions were used to examine whether the longitudinal associations of self-compassion (independent variable) with the parenting measures (dependent variable) remained significant after controlling for children’s gender and parents’ education and depressive symptoms. We controlled for these factors for the same reasons discussed in the data analysis section of study 1. Children’s gender, parents’ education, and depressive symptoms were entered at step 1 and self-compassion at step 2 (Table 6).

We also used hierarchical linear regressions to examine whether the associations of self-compassion (independent variable) with children’s internalizing and externalizing problems (dependent variable) remained significant when controlling for children’s gender and baseline internalizing or externalizing problems (time 1) and parents’ education and depressive symptoms. Children’s gender, baseline internalizing or externalizing problems (time 1), parents’ education, and depressive symptoms were entered at step 1 and self-compassion at step 2 (Table 7). These factors were considered in the analysis because child psychopathology is more likely to occur in boys, when children have earlier psychopathology, and in parents with depression and limited education (Bufferd et al. 2014; Egger and Angold 2006). Effect sizes were calculated based on adjusted *R*^2^.

All analyses were conducted separately for each parent to investigate whether consistent patterns of associations occur for mothers and fathers. The residuals in all regression analyses were examined to test the normality of distributions. Since mothers’ and fathers’ critical comments were positively skewed, these variables were inverse-transformed. We controlled for multiple comparisons using Bonferroni for mothers and fathers, separately. Four comparisons were conducted when the outcome variable was parenting and two comparisons when the outcome variable was children’s outcomes. The critical *a* values were 0.0125 and 0.025, respectively. The final sample sizes (Tables 6 and 7) included in the regression analyses were sufficiently large to detect medium effect sizes (Green 1991).

## Results

Table 3 presents participants’ characteristics. The mean age of the fathers and mothers was 39 and 36 years, respectively. The majority of participants were married or living together, had higher than average levels of education, and their ethnic background was White British. There was roughly equal numbers of boys and girls. Range, means, and standard deviations for study variables at times 1 and 2 are presented in Table 4. Table 5 presents correlations among study variables. Self-compassion correlated negatively with depressive symptoms (times 1 and 2) for mothers and fathers. Mothers’ self-compassion correlated with fewer critical (times 1 and 2) and more positive comments (time 1), fewer distressed (times 1 and 2), and more problem-focused reactions (time 1) while fathers’ self-compassion correlated with fewer distressed reactions (times 1 and 2). There were significant negative correlations of mothers’ self-compassion with child externalizing (times 1 and 2) and internalizing problems (time 1). Fathers’ self-compassion correlated with lower levels of child internalizing symptoms (times 1 and 2).

**Table 3:** Characteristics of participants (study 2)

	Fathers	Mothers	Child
Child gender at time 1			
Male *N* (%)			75(47 %)
Age (mean and *SD*) at time 1	38.8 (0.6)	36.4 (0.5)	3.9 (0.8)
Marital status^a^ at time 1			
Married or cohabitating	148 (95 %)		
Divorced, separated or single	9 (5 %)		
Parent education^b^ *N* (%) at time 1			
No qualifications, GCSE’s and A levels	52 (33 %)	49 (35 %)	
Diploma or equivalent	14 (9 %)	7 (5 %)	
Degree	43 (27 %)	44 (31 %)	
Postgraduate degree	48 (31 %)	41 (29 %)	
Ethnicity^c^ at time 1			
White British	138 (95 %)	130 (95 %)	
Number of participants at time 1	160	146	
Number of participants with depression at time 1	40 (25 %)	50 (34 %)	
Number of participants at time 2	106	98	
Number of participants with depression at time 2	33 (31 %)	37 (38 %)	

^a^Data on marital status were missing for 3 participants

^b^Data for 3 fathers’ education were missing; data for 5 mothers’ education were missing

^c^Data on ethnicity were missing for 15 fathers and 23 mothers

**Table 4:** Range, means, and standard deviations for study variables (study 2)

	Number	Range	*M* (*SD*)	Number	Range	*M* (*SD*)
	Mothers			Fathers		
Self-compassion (time 1)	120	33–119	81.61 (17.33)	133	34–122	85.15 (17.19)
Current depressive symptoms (time 1)	144	0–20	3.06 (3.55)	157	0–21	3.69 (4.44)
Current depressive symptoms (time 2)	98	0–16	2.77 (3.24)	104	0–20	3.21 (3.85)
Critical comments (time 1)	144	0–12	1.60 (2.36)	158	0–15	1.37 (2.16)
Critical comments (time 2)	93	0–13	1.74 (2.43)	105	0–9	1.54 (1.98)
Positive comments (time 1)	144	0–30	8.53 (5.14)	158	0–25	7.56 (5.10)
Positive comments (time 2)	93	0–20	8.01 (4.87)	105	0–18	7.36 (4.34)
Distressed reactions (time 1)	142	1–5.92	2.67 (.77)	149	1–4.75	2.64 (.74)
Distressed reactions (time 2)	92	1.08–5.08	2.80 (.77)	98	1.25–5	2.63 (.83)
Problem focused reactions (time 1)	142	3.50–7	5.85 (.59)	148	3–7	5.62 (.68)
Problem focused reactions (time 2)	92	2.75–7	5.73 (.69)	98	3.25–6.83	5.60 (.75)
Internalizing symptoms (time 1)	141	0–30	6.98 (5.64)	149	0–36	6.96 (5.98)
Externalizing symptoms (time 1)	142	0–29	9.86 (6.76)	148	0–40	10.46 (6.85)
Internalizing symptoms (1½ -5) (time 2)	63	0–26	6.03 (5.83)	69	0–23	5.84 (4.78)
Externalizing symptoms (1½ - 5) (time 2)	63	0–36	8.14 (6.78)	69	0–30	9.35 (6.76)
Internalizing symptoms (6–18) (time 2)	29	0–10	4.00 (2.98)	29	0–13	3.45 (3.55)
Externalizing symptoms (6–18) (time 2)	29	0–25	7.07 (6.16)	29	0–30	5.38 (6.59)

**Table 5:** Correlations of self-compassion with parenting and child outcomes for mothers and fathers (study 2)

	Mothers’ self-compassion (time 1)	Fathers’ self-compassion (time 1)
Depressive symptoms (time 1)	−0.50^b^	−0.45^b^
Depressive symptoms (time 2)	−0.43^b^	−0.54^b^
Critical comments (time 1)	−0.28^b^	−0.15
Critical comments (time 2)	−0.30^b^	−0.10
Positive comments (time 1)	0.19^a^	−0.02
Positive comments (time 2)	0.18	0.11
Distressed reactions (Time 1)	−0.43^b^	−0.36^b^
Distressed reactions (time 2)	−0.29^a^	−0.32^b^
Problem-focused reactions (time 1)	0.26^b^	0.11
Problem-focused reactions (time 2)	0.12	0.20
Internalizing symptoms (time 1)	−0.20^a^	−0.20^a^
Externalizing symptoms (time 1)	−0.29^b^	−0.18
Internalizing symptoms (time 2)	−0.20	−0.35^b^
Externalizing symptoms (time 2)	−0.31^b^	−0.15

^a^<0.05

^b^<0.01

Mothers’ and fathers’ reports on children’s internalizing (time 1: *r* = 0.64, *p* < 0.01, and time 2: *r* = 0.53, *p* < 0.01) and externalizing problems (time 1: *r* = 0.54, *p* < 0.01, and time 2: *r* = 0.63, *p* < 0.01) correlated significantly as expected. We averaged mothers’ and fathers’ z-scores for each variable and used these four mean z-scores (child internalizing and externalizing problems at time 1 and child internalizing and externalizing problems at Time 2) for all subsequent analyses.

Table 6 presents the results of the hierarchical linear regressions for parenting outcomes. After controlling for confounders (child gender, parent education, and depressive symptoms), mothers’ self-compassion was associated with fewer critical comments (*β* = −0.33, *p* < 0.05). For fathers, after controlling for confounders, self-compassion was associated with fewer distressed (*β* = −0.32, *p* < 0.01) and more problem-focused reactions (*β* = 0.29, *p* < 0.05). The effect sizes for critical comments (*f*^2^ = 0.10), distressed reactions (*f*^2^ = 0.10), and problem solving (*f*^2^ = 0.06) were small to medium.

**Table 6:** Hierarchical linear regressions showing the longitudinal associations of parents’ self-compassion with parenting after controlling for children’s gender and parents’ education and depressive symptoms (study 2)

		Critical comments (time 2)			Positive comments (time 2)			Distressed reactions (time 2)			Problem-focused reactions (time 2)	
	*N*	*ΔR* ^2^	*β*	*N*	*ΔR* ^2^	*β*	*N*	*ΔR* ^2^	*β*	*N*	*ΔR* ^2^	*β*
Mothers	71			71			69			69		
Step 1		0.13^a^			0.02			0.18^b^			0.00	
Children’s gender (0 = female, 1 = male)			−0.25^a^			−0.04			−0.23^a^			0.03
Mothers’ education (0 = no degree, 1 = degree)			−0.22			0.02			0.20			0.03
Mothers’ depressive symptoms (time 1)			0.06			−0.13			0.31^b^			0.08
Step 2		0.08^a^			0.04			0.02			0.02	
Children’s gender (0 = female, 1 = male)			−0.21			−0.07			−0.22			0.01
Mothers’ education (0 = no degree, 1 = degree)			−0.24^a^			0.04			0.19			0.04
Mothers’ depressive symptoms (time 1)			−0.09			−0.03			0.23			0.16
Mothers’ self-compassion (time 1)			−0.33^a^			0.22			−0.17			0.17
Fathers	87			87			82			82		
Step 1		0.03			0.00			0.13^a^			0.04	
Children’s gender (0 = female, 1 = male)			−0.07			−0.01			0.06			−0.18
Fathers’ education (0 = no degree, 1 = degree)			0.14			−0.07			0.21			0.10
Fathers’ depressive symptoms (time 1)			0.08			−0.05			0.28^a^			0.10
Step 2		0.00			0.01			0.08^b^			0.06^a^	
Children’s gender (0 = female, 1 = male)			−0.07			0.01			0.02			−0.14
Fathers’ education (0 = no degree, 1 = degree)			0.14			−0.10			0.29^a^			0.03
Fathers’ depressive symptoms (time 1)			0.09			0.01			0.14			0.23
Fathers’ self-compassion (time 1)			0.03			0.14			−0.32^b^			0.29^a^

When we used a research diagnosis of depression, self-compassion was associated with mothers’ critical comments (*β* = −0.25, *p* < 0.05 and distressed reactions (*β* = −0.30, *p* < 0.05). There was also a significant association between self-compassion and distressed reactions for fathers (*β* = −0.37, *p* < 0.01). When we tested for multiple comparisons, the results remained significant for fathers’ distressed reactions

^a^<0.05

^b^<0.01

In the final models, among confounders, only parent education was significant. Mothers’ higher education was associated with fewer critical comments (*β* = −0.24, *p* < 0.05) while fathers’ higher education was associated with more distressed reactions (*β* = 0.29, *p* < 0.05). When we controlled for multiple comparisons, the associations were significant for mothers’ critical comments and fathers’ distressed reactions.

When the dependent variable was children’s outcomes (Table 7), the results showed that after controlling for confounders (children’s gender, baseline problems, and mothers’ education and depressive symptoms), mothers’ self-compassion was not significantly associated with children’s outcomes. For fathers, self-compassion was inversely correlated with internalizing problems even when controlling for children’s gender, baseline emotional symptoms, and fathers’ education and depressive symptoms (*β* = −0.22, *p* < 0.05). The effect size was small to medium (*f*^2^ = 0.07). However, when we controlled for multiple comparisons, this association was no longer significant. For both parents, baseline child outcomes (time 1) were positively associated with emotional and behavioral problems at time 2 (*p* < 0.001).

**Table 7:** Hierarchical linear regressions showing the longitudinal associations of parents’ self-compassion with children’s emotional and behavioral problems at time 2 after controlling for children’s gender and baseline problems (time 1) and parents’ education and depressive symptoms (study 2)

			Externalizing problems (time 2)			Internalizing problems (time 2)
	*N*	*ΔR* ^2^	β	*N*	*ΔR* ^2^	*β*
Mothers	67			68		
Step 1		0.58^b^			0.45^b^	
Children’s gender (0 = female, 1 = male)			−0.04			−0.07
Mothers’ education (0 = no degree, 1 = degree)			−0.05			−0.16
Mothers’ depressive symptoms (time 1)			0.08			0.12
Children’s outcomes (time 1)			0.72^b^			0.59^b^
Step 2		0.00			0.00	
Children’s gender (0 = female, 1 = male)			−0.04			−0.07
Mothers’ education (0 = no degree, 1 = degree)			−0.05			−0.16
Mothers’ depressive symptoms (time 1)			0.07			0.10
Children’s outcomes (time 1)			0.72^b^			0.58^b^
Mothers’ self-compassion (time 1)			−0.008			−0.05
Fathers	72			73		
Step 1		0.61^b^			0.44^b^	
Children’s gender (0 = female, 1 = male)			−0.11			−0.11
Fathers’ education (0 = no degree, 1 = degree)			0.15			0.09
Fathers’ depressive symptoms (time 1)			0.19^a^			0.19^a^
Children’s outcomes (time 1)			0.75^b^			0.60^b^
Step 2		0.00			0.04^a^	
Children’s gender (0 = female, 1 = male)			−0.11			−0.14
Fathers’ education (0 = no degree, 1 = degree)			0.16			0.14
Fathers’ depressive symptoms (time 1)			0.16			0.10
Children’s outcomes (time 1)			0.75^b^			0.55^b^
Fathers’ self-compassion (time 1)			−0.05			−0.22^a^

When we used a research diagnosis of depression (0 = nondepressed, 1 = depressed), fathers’ self-compassion was associated with child internalizing symptoms (*β* = −0.30, *p* < 0.01). When we tested for multiple comparisons, this association remained significant

^a^<0.05

^b^<0.001

## Discussion

Study 2 suggests an association of self-compassion with parenting behaviors in parents with a history of depression. Greater self-compassion was associated with lower levels of mothers’ child-directed criticism and fathers’ distressed reactions. While the study revealed significant longitudinal associations between fathers’ self-compassion and lower levels of emotional problems among their children, these associations become nonsignificant in the regression analysis.

### General Discussion

Our findings provide support for the hypothesis that self-compassion may be associated with lower levels of mothers’ child-directed criticism and fathers’ distressed reactions. Contrary to the study hypotheses, there were no significant associations of self-compassion with parents’ positive comments and problem-focused responses. While correlational analysis revealed significant negative associations of fathers’ self-compassion with children’s internalizing problems, these associations became nonsignificant in the regression analysis. Before discussing the implications of these findings, we review methodological issues raised by the work. A strength is that parenting was examined using observations, questionnaires, and interviews. Both studies included samples of parents with a research diagnosis of a history of depression and study 2 included a sufficient sample of fathers for an analysis stratified by parental gender. Triangulation across two samples allowed us to test whether self-compassion was associated with parenting behaviors in two samples of parents, including some with current and/or a history of depression for whom parenting can be challenging. However, both studies also had limitations. Study 1 was cross-sectional and study 2, while having more than one time point, was correlational; therefore, no causal inferences can be drawn. In both studies, the sample included a relatively small number of parents with a history of depression, and so we may have lacked sufficient power to identify all important relationships of parenting with self-compassion. Furthermore, there were only seven fathers with a current depressive illness in study 2, so we could not examine whether the associations were more evident in currently depressed versus all parents with a history of depression. The sample was more educated than a general community sample, which limits generalizability. Effect sizes were small to medium, suggesting that self-compassion is one out of many other factors that predict these parenting behaviors. Study 2 recruited mothers through fathers’ participation, which may have biased the findings. Finally, the large number of statistical tests conducted increased the possibility of type I error, which we addressed by correcting the level of significance using Bonferroni. Nonetheless, the pattern of findings is similar across both samples, which suggests that the association of self-compassion with these parenting attributes is likely to be replicable.

Overall, the findings provide some support for our hypothesis that individual differences in self-compassion are associated with fewer fathers’ distressed reactions to their children’s negative emotions. Previous studies have found that parents who use positive responses to cope with their children’s negative emotions have children who are better able to regulate their emotions than parents who react negatively and increase the child’s arousal (Eisenberg et al. 1996; Fabes et al. 2001, 2002). Parents who display fewer distressed reactions may be more able to validate their children’s emotions and convey the message that these emotions are understandable, while children may learn to express rather than suppress these emotions (Fabes et al. 2001). In turn, children may become better able to regulate these emotions in similar situations, which may decrease their risk of emotional problems. When we adjusted for multiple comparisons on multivariate analysis, the link of self-compassion with fathers’ problem-focused responses became nonsignificant. Nonetheless, our findings provide promising avenues for future research using larger scale and experimental/interventional designs.

Our study suggests that higher levels of self-compassion are associated with lower levels of maternal criticism. Previous studies have found that depressed parents are more critical and less positive (Barnes et al. 2007; Psychogiou et al. 2013; Rogosch et al. 2004) and that high EE is associated with children’s emotional and behavioral problems (e.g., Cartwright et al. 2011; Nelson et al. 2003; Peris and Baker 2000; Psychogiou et al. 2007). Although our study could not explore the mechanisms that may explain this association, it is possible that greater self-compassion may enable parents to recognize, be kind toward and de-center from negative thoughts and the impulse to react with criticism. It enables parents to respond to both their negative thoughts and their children’s needs more adaptively. The pattern of associations across self-compassion and parenting behaviors, both positive and negative, converges on this explanation. This could be tested empirically among parents at risk for depression randomized to interventions that either seek to increase self-compassion or build resilience through an alternative mechanism (e.g., cognitive therapy). If future research suggests that increasing self-compassion reduces EE, interventions that can target either or both might improve parent and child outcomes.

Significant correlations between self-compassion and lower levels of children’s emotional and behavioral problems were found. While it should be interpreted with caution, study 2 indicates that fathers’, but not mothers’, self-compassion was significantly associated with lower levels of internalizing problems among their children at time 2 when controlling for depressive symptoms and earlier child internalizing symptoms. However, when we controlled for multiple comparisons, this association became nonsignificant. Given the significant longitudinal associations in the correlational analysis, perhaps an important direction for future research is to establish if parents’ self-compassion is related to lower levels of child psychopathology and if so, how and why such an association occurs. As we discussed earlier, children of parents with greater self-compassion may receive more positive parenting or they may observe and adopt a parent’s capacity to self-soothe in difficult times. Gilbert et al. (2006) have suggested that children exposed to criticism may develop self-evaluative scripts viewing their parents as hostile and become oversensitive and vulnerable to actual and perceived rejection. In contrast, children not exposed to criticism may develop a sense of being worthy of support and in times of stress, they may be better able to self-soothe. An important direction for future research is to test these hypotheses.

The findings have potential implications for future research and interventions that target depressed parents and their children. Interventions that aim to increase parents’ self-compassion may result in decreased parental depressive symptoms and/or improved child mental health. Kuyken et al. (2010) found that increases in self-compassion through mindfulness-based cognitive therapy among people with a history of depression were associated with fewer depressive symptoms 15 months later. In a qualitative study, parents who took part in mindfulness-based cognitive therapy reported that they were better able to recognize their own needs and regulate their emotions. They also reported that they were more emotionally available and empathetic toward their children (Bailie et al. 2012). Given that fathers’ self-compassion was significantly associated with parenting, parenting programs should encourage the attendance of both parents.

In the future, these findings need to be replicated with other populations (e.g., parents with bipolar, schizophrenia, or substance abuse/dependence disorders) to examine their specificity to depression and with other dimensions of parenting behaviors. There is also a need for research designs that include both depressed mothers and fathers to uncover similarities and differences in the associations of self-compassion with parenting.

## References

[CR1] Achenbach T, Rescorla LA (2000). Manual for the ASEBA Preschool Forms & Profiles.

[CR2] Arnau RC, Meagher MW, Norris MP, Bramson R (2001). Psychometric evaluation of the Beck Depression Inventory-II with primary care medical patients. Health Psychology.

[CR3] Bailie C, Kuyken W, Sonnenberg S (2012). The experiences of parents in mindfulness-based cognitive therapy. Clinical Child Psychology and Psychiatry.

[CR4] Barnard LK, Curry JF (2011). Self-compassion: conceptualizations, correlates, and interventions. Review of General Psychology.

[CR5] Barnes, J., Ram, B., Leach, P., Altmann, L., Sylva, K., Malmberg, L. E., … & Team, T. F. (2007). Factors associated with negative emotional expression: a study of mothers of young infants. *Journal of Reproductive and Infant Psychology. 25*:122–138.

[CR6] Basco MR, Bostic JQ, Davies D, Rush AJ, Witte B, Hendrickse W, Barnett V (2000). Methods to improve diagnostic accuracy in a community mental health setting. American Journal of Psychiatry.

[CR7] Beck AT, Haigh EAP (2014). Advances in cognitive theory and therapy: the generic cognitive model. Annual Review of Clinical Psychology.

[CR8] Beck AT, Steer RA, Brown GK (1996). The Beck Depression Inventory-Second Edition.

[CR9] Breines JG, Thoma MV, Gianferante D, Hanlin L, Chen X, Rohleder N (2014). Self-compassion as a predictor of interleukin-6 response to acute psychosocial stress. Brain, Behavior, and Immunity.

[CR10] Bufferd SJ, Dougherty LR, Olino TM, Dyson MW, Laptook RS, Carlson GA, Klein DN (2014). Predictors of the onset of depression in young children: a multi‐method, multi‐informant longitudinal study from ages 3 to 6. Journal of Child Psychology and Psychiatry.

[CR11] Campbell SB (1995). Behavior problems in preschool children: a review of recent research. Journal of Child Psychology and Psychiatry.

[CR12] Cartwright, K. L., Bitsakou, P., Daley, D., Gramzow, R. H., Psychogiou, L., Simonoff, E., … & Sonuga-Barke, E. J. (2011). Disentangling child and family influences on maternal expressed emotion toward children with attention-deficit/hyperactivity disorder. *Journal of the American Academy of Child and Adolescent Psychiatry. 50*:1042–1053. 10.1016/j.jaac.2011.07.006 21961778

[CR13] Connell AM, Goodman SH (2002). The association between psychopathology in fathers versus mothers and children’s internalizing and externalizing behavior problems: a meta-analysis. Psychological Bulletin.

[CR14] Dadds MR, Mullins MJ, McAllister RA, Atkinson E (2003). Attributions, affect, and behavior in abuse-risk mothers: a laboratory study. Child Abuse and Neglect.

[CR15] Daley D, Sonuga‐Barke EJS, Thompson M (2003). Assessing expressed emotion in mothers of preschool AD/HD children: psychometric properties of a modified speech sample. British Journal of Clinical Psychology.

[CR16] Davis RN, Davis MM, Freed GL, Clark SJ (2011). Fathers’ depression related to positive and negative parenting behaviors with 1-year-old children. Pediatrics.

[CR17] Davis-Kean PE (2005). The influence of parent education and family income on child achievement: the indirect role of parental expectations and the home environment. Journal of Family Psychology.

[CR18] Diedrich A, Grant M, Hofmann SG, Hiller W, Berking M (2014). Self-compassion as an emotion regulation strategy in major depressive disorder. Behaviour Research and Therapy.

[CR19] Dix T, Gershoff ET, Meunier LN, Miller PC (2004). The affective structure of supportive parenting: depressive symptoms, immediate emotions, and child-oriented motivation. Developmental Psychology.

[CR20] Egger HL, Angold A (2006). Common emotional and behavioral disorders in preschool children: presentation, nosology, and epidemiology. Journal of Child Psychology and Psychiatry.

[CR21] Eisenberg N, Fabes RA (1994). Mothers’ reactions to children’s negative emotions: relations to children’s temperament and anger behavior. Merrill-Palmer Quarterly.

[CR22] Eisenberg N, Fabes RA, Murphy BC (1996). Parents’ reactions to children’s negative emotions: relations to children’s social competence and comporting behavior. Child Development.

[CR23] Fabes, R.A., Eisenberg, N., & Bernzweig, J. (1990). *The Coping with Children’s Negative Emotions Scale: Procedures and scoring*. Arizona State University.

[CR24] Fabes RA, Leonard SA, Kupanoff K, Martin CL (2001). Parental coping with children’s negative emotions: relations with children’s emotional and social responding. Child Development.

[CR25] Fabes RA, Poulin RE, Eisenberg N, Madden-Derdich DA (2002). The Coping with Children’s Negative Emotions Scale (CCNES): psychometric properties and relations with children’s emotional competence. Marriage & Family Review.

[CR26] Feldman C, Kuyken W (2011). Compassion in the landscape of suffering. Contemporary Buddhism.

[CR27] Gilbert P, Baldwin MW, Irons C, Baccus JR, Palmer M (2006). Self-criticism and self-warmth: an imagery study exploring their relation to depression. Journal of Cognitive Psychotherapy.

[CR28] Goodman SH (2007). Depression in mothers. Annual Review of Clinical Psychology.

[CR29] Goodman SH, Gotlib IH (1999). Risk for psychopathology in the children of depressed mothers: a developmental model for understanding mechanisms of transmission. Psychological Review.

[CR30] Goodman SH, Rouse MH, Connell AM, Broth MR, Hall CM, Heyward D (2011). Maternal depression and child psychopathology: a meta-analytic review. Clinical Child and Family Psychology Review.

[CR31] Gorman, L. L., O’Hara, M. W., Figueiredo, B., Hayes, S., Jacquemain, F., Kammerer, M. H., … & Sutter-Dallay, A. L. (2004). Adaptation of the Structured Clinical Interview for DSM–IV Disorders for assessing depression in women during pregnancy and post-partum across countries and cultures. *The British Journal of Psychiatry. 184*(46):s17-s23. 10.1192/bjp.184.46.s17 14754814

[CR32] Green SB (1991). How many subjects does it take to do a regression analysis?. Multivariate Behavioral Research.

[CR33] Kabat-Zinn, M., & Kabat-Zinn, J. (1998). Everyday blessings: The inner work of mindful parenting. *Hyperion*.

[CR34] Kim-Cohen J, Caspi A, Moffitt TE, Harrington H, Milne BJ, Poulton R (2003). Prior juvenile diagnoses in adults with mental disorder: developmental follow-back of a prospective-longitudinal cohort. Archives of General Psychiatry.

[CR35] Klimecki OM, Leiberg S, Lamm C, Singer T (2013). Functional neural plasticity and associated changes in positive affect after compassion training. Cerebral Cortex.

[CR36] Klimecki OM, Leiberg S, Ricard M, Singer T (2013). Differential pattern of functional brain plasticity after compassion and empathy training. Social Cognitive and Affective Neuroscience.

[CR37] Krieger T, Altenstein D, Baettig I, Doerig N, Holtforth MG (2013). Self-compassion in depression: associations with depressive symptoms, rumination, and avoidance in depressed outpatients. Behavior Therapy.

[CR38] Kuyken, W., Watkins, E., Holden, E., White, K., Taylor, R. S., Byford, S., … & Dalgleish, T. (2010). How does mindfulness-based cognitive therapy work? *Behaviour Research and Therapy. 48*:1105–1112. 10.1016/j.brat.2010.08.003 20810101

[CR39] Lépine JP, Briley M (2011). The increasing burden of depression. Neuropsychiatric Disease and Treatment.

[CR40] Lobbestael J, Leurgans M, Arntz A (2010). Inter-rater reliability of the Structured Clinical Interview for DSM-IV Axis I Disorders (SCID I) and Axis II Disorders (SCID II). Clinical Psychology & Psychotherapy.

[CR41] Lovejoy MC, Graczyk PA, O’Hare E, Neuman G (2000). Maternal depression and parenting behavior: a meta-analytic review. Clinical Psychology Review.

[CR42] Maniadaki K, Sonuga‐Barke E, Kakouros E (2005). Parents’ causal attributions about attention deficit/hyperactivity disorder: the effect of child and parent sex. Child: Care, Health and Development.

[CR43] Mann, J., Kuyken, W., O’Mahen, H., Ukoumunne, O., Evans, A., & Ford, T. (under review). Manual development and pilot randomised controlled trial of mindfulness-based cognitive therapy versus usual care for parents with a history of depression. *Mindfulness.* 10.1007/s12671-016-0543-7 PMC5010613 27642371

[CR44] Matias, C., Scott, S., & O’Connor, T.G. (2006). *Coding of attachment-related parenting (CARP).* Unpublished manuscript, Institute of Psychiatry, King’s College London, United Kingdom.

[CR45] Nath S, Russell G, Ford T, Kuyken W, Psychogiou L (2015). Postnatal paternal depressive symptoms associated with fathers’ subsequent parenting: findings from the Millennium Cohort Study. British Journal of Psychiatry.

[CR46] Neff KD (2003). Self-compassion: an alternative conceptualization of a healthy attitude toward oneself. Self and Identity.

[CR47] Neff KD (2003). The development and validation of a scale to measure self-compassion. Self and Identity.

[CR48] Neff KD, Kirkpatrick KL, Rude SS (2007). Self-compassion and adaptive psychological functioning. Journal of Research in Personality.

[CR49] Nelson DR, Hammen C, Brennan PA, Ullman JB (2003). The impact of maternal depression on adolescent adjustment: the role of expressed emotion. Journal of Consulting and Clinical Psychology.

[CR50] O’Connor TG, Matias C, Futh A, Tantam G, Scott S (2013). Social learning theory parenting intervention promotes attachment-based caregiving in young children: Randomized clinical trial. Journal of Clinical Child and Adolescent Psychology.

[CR51] Patterson G (1982). Coercive family process.

[CR52] Paulson JF, Dauber S, Leiferman JA (2006). Individual and combined effects of postpartum depression in mothers and fathers on parenting behavior. Pediatrics.

[CR53] Peris TS, Baker BL (2000). Applications of the expressed emotion construct to young children with externalizing behavior: Stability and prediction over time. Journal of Child Psychology and Psychiatry.

[CR54] Psychogiou L, Daley DM, Thompson MJ, Sonuga-Barke EJ (2007). Mothers’ expressed emotion toward their school-aged sons. European Child and Adolescent Psychiatry.

[CR55] Psychogiou L, Netsi E, Sethna V, Ramchandani PG (2013). Expressed emotion as an assessment of family environment with mothers and fathers of one-year old children. Child: Care, Health and Development.

[CR56] Ramchandani P, Psychogiou L (2009). Paternal psychiatric disorders and children’s psychosocial development. The Lancet.

[CR57] Ramchandani P, Stein A, Evans J, O’Connor TG (2005). Paternal depression in the postnatal period and child development: a prospective population study. The Lancet.

[CR58] Rogosch FA, Cicchetti D, Toth SL (2004). Expressed emotion in multiple subsystems of the families of toddlers with depressed mothers. Development and Psychopathology.

[CR59] Spitzer RL, Kroenke K, Williams JB, Patient Health Questionnaire Primary Care Study Group (1999). Validation and utility of a self-report version of PRIME-MD: the PHQ primary care study. JAMA.

[CR60] Weitzman M, Rosenthal DG, Liu YH (2011). Paternal depressive symptoms and child behavioral or emotional problems in the United States. Pediatrics.

[CR61] Williams MJ, Dalgleish T, Karl A, Kuyken W (2014). Examining the factor structures of the Five Facet Mindfulness Questionnaire and the Self-Compassion Scale. Psychological Assessment.

[CR62] Wilson S, Durbin CE (2010). Effects of paternal depression on fathers’ parenting behaviors: a meta-analytic review. Clinical Psychology Review.

